# Clinicopathological utility of human epidermal growth factor receptor 2 (HER2)-heterogeneity for next-generation treatments of triple-negative breast cancer

**DOI:** 10.18632/oncotarget.28007

**Published:** 2021-10-26

**Authors:** Sasagu Kurozumi, Ayaka Katayama, Ken Shirabe, Jun Horiguchi, Emad A. Rakha

**Keywords:** breast cancer, HER2 low, triple negative, heterogeneity

In recent years, developments in the diagnosis and treatment modalities of breast cancer (BC) have greatly improved the prognosis and outcome. Systemic therapy approaches in BC include targeted therapy, cytotoxic chemotherapy, immunotherapy and other biological therapies and it is based on a set of prognostic clinicopathological variables and few molecular predictive variables. In routine practice, the most important predictive variables in BC are hormone receptor (HR) including oestrogen receptor (ER) and progesterone receptor (PR), and human epidermal growth factor receptor 2 (HER2). HR positive BC patients are candidate for endocrine therapy whereas HER2 positive BC patients are typically candidate for anti-HER2 targeted therapy combined with chemotherapy as these tumours are typically aggressive. These therapies have resulted in significant improved of BC outcome with more than 50% reduction in the early and late recurrences and mortalities. However, approximately 15% of BC lack the expression of HR and HER2 and these tumours are classified as Triple-negative BC (TNBC). TNBC not only lack the benefits of endocrine therapy or anti-HER2 therapy but are also characterised by aggressive clinicopathological features, such as high grade and suffer from early recurrences and high mortality.

In recent treatment strategies of triple-negative BC, novel molecular targeting drugs, such as PARP-1 inhibitor and immune checkpoint inhibitors, are attempted; however, the response remains limited and other effective therapeutic options are yet to be determined. Accordingly, we need to elucidate the molecular biological characteristics of TNBC to identify new target molecules and novel therapeutic strategies.

Similar to TNBC, HER2-positive BC has aggressive clinical phenotypes and unfavourable prognosis. HER2 is a transmembrane receptor tyrosine kinase that mediates several functions such as growth, differentiation and survival of BC cells. Initially in the year 2000, trastuzumab was developed as a molecular targeting agent against HER2 protein and clinical trials have demonstrated its effectiveness in the treatment of HER2-positive BC [1]. Currently, several types of HER2-targeting therapeutic agents are available for the treatment of HER2-positive BC that have drastically improved the prognosis [2, 3]. In actual clinical practice and clinical research, HER2 testing methods primarily include immunohistochemistry (IHC) and *in situ* hybridisation (ISH) to demonstrate protein overexpression, which is typically a reflection of *HER2* gene amplification, or evidence of *HER2* gene amplification either alone or in BC with equivocal HER2 protein expression. HER2 overexpression and/or gene amplification is detected in approximately 15% of BC and these tumours are the only candidate for anti-HER2 therapies whereas patients with BC featuring low level of HER2 protein expression or lack evidence of gene amplification are not offered such treatments. These tumours were classified as HER2 negative BC to indicate that those patients are not eleigible for anti-HER2 therapies. However, these HER2 negative tumours comprise a heterogeneous group of BC including HR+ and HR- (TNBC) and tumours without any protein expression (IHC score 0 [4]) and those with low levels of protein expression (IHC score 1+ and 2+ without evidence of gene amplification [4]). The later groups are now called as HER2 low BC [5]. HER2 low BC comprises approximately 50% of BC and 35% of TNBC (unpublished data).

Although the effects of existing anti-HER2 treatment are limited in the low-HER2 expression group, it has been reported that the new anti-HER2 antibody-drug conjugates (ADC) such as trastuzumab deruxtecan [6], is effective in HER2-low BC. The emergence and the success of the ADC can be considered as one of the most promising new tools for the selective ablation of BC that express proteins regardless of the gene status. Several ADCs have already received regulatory approval and many others are in different phases of clinical development. ADCs comprise a monoclonal antibody against a tumor-associated antigen, a covalent linker, and a cytotoxic payload [7]. In recent years, it has been shown that HER2 protein can be used as a tumour associated antigen. In HER2 expressing BC cells, trastuzumab deruxtecan is internalized upon binding to the cognate HER2 antigen, and the cytotoxic payload is released, causing cell death. Such targeted delivery of cytotoxins to BC cells improve the therapeutic efficacy of the chemotherapeutic agents. Therefore, the effects are also seen in HER2 negative BC cells surrounding HER2-positive cells (bystander effect), and it has thus drawn attention as an effective new therapeutic agent for low-HER2 BC including TNBC [8]. Currently, two randomized, phase3 trials with trastuzumab deruxtecan in low-HER2 BC are ongoing [5].

**Figure 1:**
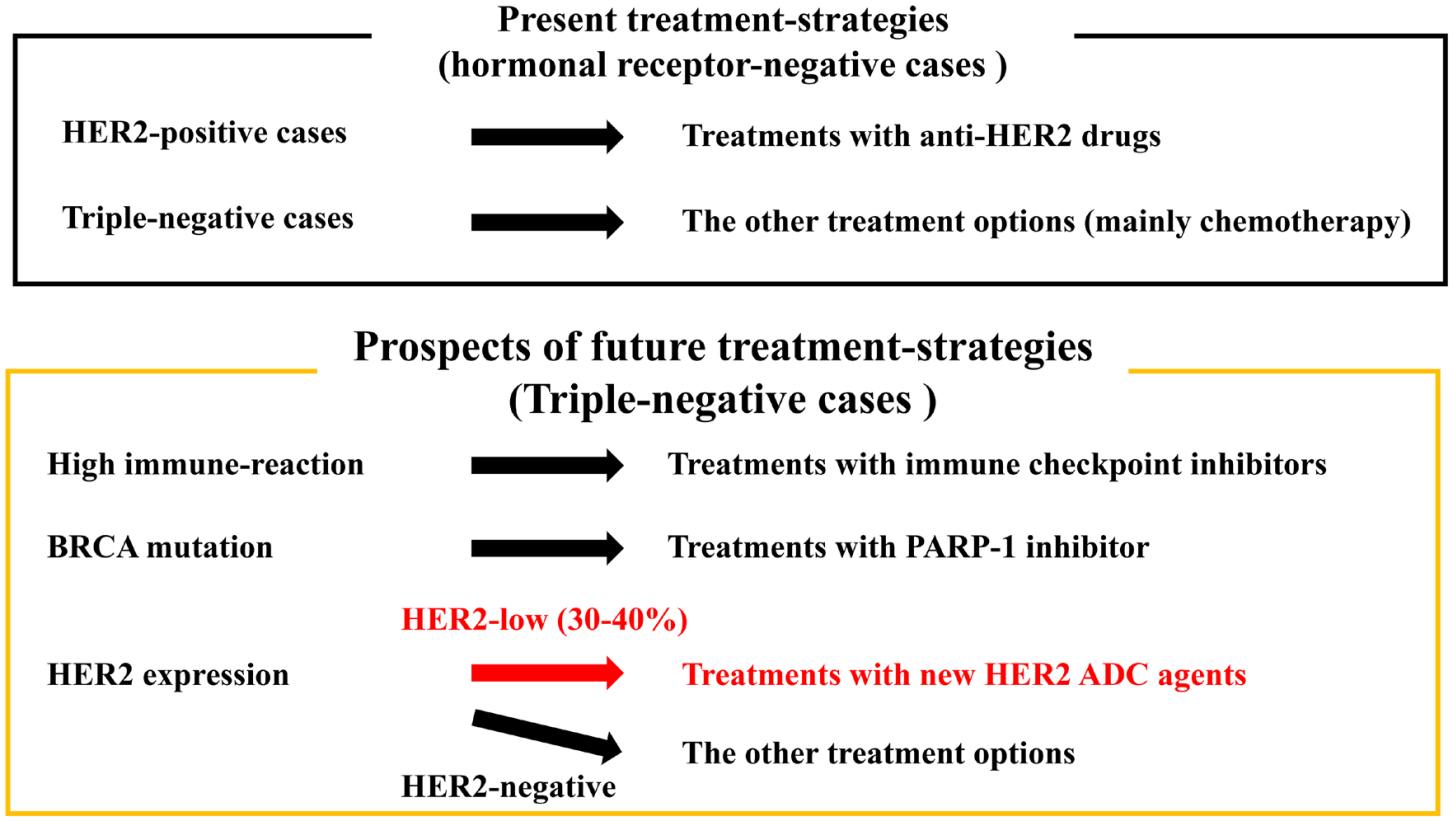
Prospects on the changing of treatment-strategies based on HER2-expression for triple-negative cases. In a present clinical practice, anti-HER2 treatments cannot be used for breast cancer with triple-negative type. Future translational research and clinical trial investigating the ability of new anti-HER2 antibody-drug conjugates (ADC) agents to HER2-heterogeneity type may lead to new treatment-strategies for triple-negative breast cancer.

However, the introduction of the HER2 low concept and the success of ADC in HER2 expressing TNBC have raised many issues including the distinction between score 0 and score 1+ in routine practice and refinement of the definition and the impact of HER2 heterogeneity, which is more obvious in the HER2 low more than in HER2 negative (IHC score 0) or HER2 positive (IHC score 3+) BC. On the other hand, cases are categorized as HER2 heterogeneous when at least two different types of HER2 gene and protein status combinations were observed. As this new concept for HER2 category, low-HER2 cases might be categorized into HER2-heterogeneity or HER2-non-heterogeneity type. In recent years, it has been suggested that there is a high incidence of HER2-heterogeneity in HER2-negative patients, which contributes to poor prognosis. In our study of HER2-negative BC, we found significantly poorer prognosis in the group presenting HER2-heterogeneity, with a particularly greater significant difference in TNBC [9]. In future, in order to use such agents in actual clinical practice and to refine the application of such treatment, we may need to establish a new method of determining HER2 expression based on HER2-heterogeneity. Discussions on the relationship between tumour heterogeneity and the effects of new molecular targeting agents might become more active with regard to BC (Figure 1).
